# Identification of key candidate biomarkers for severe influenza infection by integrated bioinformatical analysis and initial clinical validation

**DOI:** 10.1111/jcmm.16275

**Published:** 2021-01-14

**Authors:** Shuai Liu, Zhisheng Huang, Xiaoyan Deng, Xiaohui Zou, Hui Li, Shengrui Mu, Bin Cao

**Affiliations:** ^1^ China‐Japan Friendship Hospital National Clinical Research Center for Respiratory Diseases Clinical Center for Pulmonary Infections Capital Medical University Beijing China; ^2^ Department of Pulmonary and Critical Care Medicine Center for Respiratory Diseases China‐Japan Friendship Hospital Beijing China; ^3^ Institute of Respiratory Medicine Chinese Academy of Medical Sciences Peking Union Medical College Beijing China; ^4^ Tsinghua University‐Peking University Joint Center for Life Sciences Tsinghua University Beijing China

**Keywords:** differentially expressed genes, hub genes, network analysis, neutrophils, severe influenza, weighted gene co‐expression network analysis

## Abstract

One of the key barriers for early identification and intervention of severe influenza cases is a lack of reliable immunologic indicators. In this study, we utilized differentially expressed genes screening incorporating weighted gene co‐expression network analysis in one eligible influenza GEO data set (GSE111368) to identify hub genes associated with clinical severity. A total of 10 genes (*PBI*, *MMP8*, *TCN1*, *RETN*, *OLFM4*, *ELANE*, *LTF*, *LCN2*, *DEFA4* and *HP*) were identified. Gene set enrichment analysis (GSEA) for single hub gene revealed that these genes had a close association with antimicrobial response and neutrophils activity. To further evaluate these genes' ability for diagnosis/prognosis of disease developments, we adopted double validation with (a) another new independent data set (GSE101702); and (b) plasma samples collected from hospitalized influenza patients. We found that 10 hub genes presented highly correlation with disease severity. In particular, *BPI* and *MMP8* encoding proteins in plasma achieved higher expression in severe and dead cases, which indicated an adverse disease development and suggested a frustrating prognosis. These findings provide new insight into severe influenza pathogenesis and identify two significant candidate genes that were superior to the conventional clinical indicators. These candidate genes or encoding proteins could be biomarker for clinical diagnosis and therapeutic targets for severe influenza infection.

## 1 INTRODUCTION

Seasonal influenza infection is associated with 84 000‐92 000 deaths every year in China.^1^ Some special population, particularly the elderly and immunodeficient people, are more likely to develop into refractory hypoxaemia and even respiratory failure.^2^ To assess illness status timely and predict disease progression correctly is important for influenza patient‐management, but remains a challenge. Circulating T lymphocytes counts and virus‐specific CD4^+^ and CD8^+^ T cells levels, as detectable markers, have been identified as the indicators related to infection severity.^3^, ^4^, ^5^ In addition, procalcitonin and C‐reactive protein, as widely used biomarkers in clinical practice, potentially assist in the identification from alone influenza invasion to secondary bacterial or fungi infections that follow and predict hospital mortality.^6^, ^7^ These studies, however, consisted of small sample sizes and miscellaneous factors, which could be partial and inadequate to reflect illness status or predict disease progression. Given higher morbidity and mortality in severe influenza, it is highly demanded to further investigate the molecular mechanisms of severe influenza procession, discover additional reliable biomarkers for early effective clinical diagnosis and therapy.

In recent years, transcriptomics studies of circulating leucocytes provided a unifying framework to assess host response at gene expression levels and these findings showed that host response displayed distinctively changes across the full range of healthy, moderate and severe influenza infection.^8^, ^9^, ^10^, ^11^ And many screened host factors were closely associated with the progression of severe influenza, which may assist in the discrimination of different immune response signatures between severe influenza and others.^12^ However, owing to samples heterogeneity and sampling differences, different technology platforms and analysis strategy usage in individual studies, it is difficult to perform statistical analysis and extract valuable information. Therefore, the integrated bioinformatics methods combining with expression profiling techniques could provide comprehensive and valuable clues to study the molecular pathogenesis of influenza infection and identify innovative biomarkers.

Our study aimed to identify candidate biological markers to predict severe illness progression and to discover underlying therapeutic targets of severe influenza. Firstly, one microarray data set GSE111368 ^9^ from NCBI‐Gene Expression Omnibus database (NCBI‐GEO) were analysed to identify differentially expressed genes (DEGs) between influenza patients and matched controls. And the data set was analysed to find key modules associated with clinical severity using weighted gene co‐expression network analysis (WGCNA) as well. Secondly, 43 overlapped genes from two analysis results were identified to further perform Gene Ontology (GO) and Kyoto Encyclopedia of Genes and Genomes (KEGG) analysis. And then, these genes were subjected to protein‐protein interaction (PPI) network construction and modular analysis. Lastly, four seldomly reported hub genes, *LCN2*, *BPI*, *ELANE* and *MMP8*, were selected to explore their potential biological functions with gene set enrichment analysis (GSEA) and to assess clinical value associated with disease severity or illness outcome. Overall, these findings will contribute to identifying more reliable biomarkers for early diagnosis and prognosis, or conducting accurate host‐targeted therapy after influenza infection.

## 2 MATERIALS AND METHODS

### 2.1 Ethics statement

Experiments involving human participants were conducted according to the Declaration of Helsinki and approved by the China‐Japan Friendship Hospital Ethics Committee (approval no. 2018‐120‐K86) in accordance with its guidelines for the protection of human volunteers. The participants provided their written informed consent to participate in this study.

### 2.2 Clinical samples

Whole‐blood samples were obtained from influenza patients who were admitted to China‐Japan friendship hospital, and plasma were isolated. Severe influenza was defined as a severe influenza pneumonitis and hypoxic respiratory failure that receives invasive mechanical ventilation and (or) emergency extracorporeal membrane oxygenation; Moderate influenza was defined as a significant flu‐like symptomatic disease with or without supplemental oxygen therapy (including nasal high‐flow oxygen therapy and (or) non‐invasive mechanical ventilatory support). Twenty‐five peripheral blood samples of healthy donors with no history of influenza infection were used as controls. A total of 63 influenza patients were recruited in 2017‐2018 and 2019‐2020, 34 were classified as ‘Moderate’ and 29 as ‘Severe’ according to criteria described above. Sufficient blood samples were screened and collected in our study. Flow chart of enrolled patients was shown in Figure S6. Influenza patients enrolled had at least one comorbidity. For blood tests performed at admission, severe patients had a lower proportion of lymphocytes (*P* = 0.0252) and a higher proportion of neutrophil (*P* = 0.0005) than moderate patients. 24 patients (70%) required supplemental oxygen in the moderate group. 26% (9/34) and 96% (28/29) of the patients in the moderate and severe group were admitted to the intensive care unit, respectively. The hospital mortality rates in severe cases of influenza pneumonitis were 48% (14/29). Cohort characteristics are listed in Table 1.

**TABLE 1:** Demographics and clinical characteristics of influenza patients

	Total (N = 63)	Moderate (N = 34)	Severe (N = 29)	*P* value
Gender				0.5074
Female	30 (48%)	18 (53%)	12 (41%)	
Male	33 (52%)	16 (47%)	17 (59%)	
Age/y (mean (SD))	55.88 (14.52)	59.00 (11.23)	52.24 (17.11)	0.0753
Flu virus strains				0.0654
Influenza type A	41 (65%)	25 (74%)	16 (55%)	
Influenza type B	20(32%)	7(20%)	13(45%)	
Influenza type A and B	2 (3%)	2 (6%)	0 (0%)	
Comorbidity				
Pneumonia disease	52 (82%)	23 (67%)	29 (100%)	0.0023
Cancer	3 (4%)	3 (8%)	0 (0%)	0.2427
Hypertension	20 (31%)	12 (35%)	8 (27%)	0.7012
Diabetes	15 (23%)	6 (17%)	9 (31%)	0.3437
Laboratory tests (mean (SD))				
Total leucocytes (×10^9^/L)	9.68 (6.34)	8.80 (5.80)	10.70 (6.88)	0.1949
Neutrophil (×10^9^/L)	8.01 (5.88)	6.73 (5.01)	9.51 (6.53)	0.0557
Proportion of neutrophil (%)	80.25 (13.56)	74.71 (15.63)	86.76 (6.16)	0.0005
Lymphocytes (×10^9^/L)	0.92 (0.57)	1.03 (0.66)	0.80 (0.41)	0.2639
Proportion of lymphocytes (%)	13.36 (12.09)	15.55 (11.12)	10.78 (12.85)	0.0252
Respiratory support				
Extracorporeal membrane oxygenation	12 (19%)	0 (0%)	12 (41%)	0.0001
Invasive mechanical ventilation	29 (46%)	0 (0%)	29 (100%)	0.0000
Non‐invasive mechanical ventilatory support	31 (49%)	16 (47%)	15 (51%)	0.9073
Require supplemental oxygen	53 (84%)	24 (70%)	29 (100%)	0.0011
Outcome				
Hospitalization	63 (100%)	34 (100%)	29 (100%)	0.5287
Admission to ICU	37 (58%)	9 (26%)	28 (96%)	0.0000
Death	14 (22%)	0 (0%)	14 (48%)	0.0000

Note

Data are mean (SD) or n (%). *P* values were calculated by Student's *t* test, Mann‐Whitney *U* test, chi‐square test or Fisher's exact test, as appropriate.

Abbreviation: ICU, intensive care unit.

### 2.3 Microarray data information

The NCBI‐GEO (http://www.ncbi.nlm.nih.gov/geo) is a public data storage of microarray profiles and next‐generation sequencing. We screened and downloaded data sets from GEO. The selection criteria were as follows: (a) Inclusion of gene expression profiles of influenza patients and healthy donor's blood samples, convalescent phase samples were excluded; (b) Influenza patients were diagnosed by reverse transcription polymerase chain‐reaction testing of respiratory tract sample; (c) Influenza patients had severity classification, and criteria were generally similar; (d) Data sets contained a minimum of 10 influenza patients and healthy donors' blood samples. According to the above criteria, two gene expression profiles GSE111368 and GSE101702 were obtained. The microarray data of GSE111368 included 109 influenza patients and 130 healthy controls. 179 whole‐blood samples were collected from 109 enrolled patients. An outlier sample (GSM3029336) was removed according to value of genes' expression. The microarray data of GSE101702 included 107 influenza patients and 52 healthy controls. 107 whole‐blood samples were collected from 107 enrolled patients. The two data sets performed the same diagnostic criteria and defined severe or milder illness status based on whether invasive mechanical ventilation was used or not. GSE111368 was used to screen DEGs and construct WGCNA for this study. GSE101702 was used for validation of hub genes. A flow chart of this study was shown in Figure S1.

### 2.4 Identification of DEGs and functional enrichment analyses

We downloaded the matrix files of two data sets from GEO. The R package ‘limma’ was performed for DEGs identifying between influenza samples and healthy samples. Cut‐off criteria for screening DEGs were false discovery rate < 0.05 and |log_2_fold change| ≥ 1. GO enrichment and KEGG pathway analyses were conducted using the R package ‘clusterprofiler’. GO terms or KEGG pathways with adjusted *P* < 0.05 were considered statistically significant. GO terms consisted of three aspects: biological process (BP), cellular component (CC) and molecular function (MF).

### 2.5 Analysis of hub genes expression and immune cells

To quantify the difference of immune cells between influenza patients and healthy donors, we used the x‐Cell tool (http://xcell.ucsf.edu/) to evaluate 34 immune cell types, which is a gene signature‐based method that integrates the advantages of gene set enrichment with deconvolution approaches. We utilized ‘ggstatsplot’ (R package, https://cran.r‐project.org/web/packages/ggstatsplot/) to investigate the correlation between the hub genes expression and immune cells.

### 2.6 Gene set enrichment analysis

We performed GSEA analysis between influenza and healthy samples with the R package ‘clusterprofiler’.^13^ We also used GSEA analysis to explore biological function of hub genes. Based on correlation coefficient between each hub gene and other genes, influenza samples were divided into two groups (positive correlation vs negative correlation). *P* < 0.05 was regarded as statistically significant. Annotated gene sets ‘c2.cp.kegg.v7.1.symbols.gmt’ and ‘c5.bp. v7.1.symbols.gmt’ were selected as the reference gene sets.

### 2.7 Weighted co‐expression network analysis

We extracted 3488 genes (according to variance) to construct a weight co‐expression network using the R package ‘WGCNA’.^14^ The adjacency matrix was converted into topological overlap matrix when the power of *β* = 9 (*R* ^2^ = 0.851). Then, genes were categorized into different modules with a module minimum size cut‐off of 30. Similar modules were merged together with a height cut‐off of 0.25. The module with the highest correlation with clinical traits was selected to explore its biological function through GO and KEGG analyses.

### 2.8 Hub genes detection

The genes with the most clinical correlation were defined as hub genes. Firstly, hub genes were screened according to the criteria that gene significance (GS) > 0.5 and module membership (MM) > 0.8 in the most significant module. Then, we identified common genes through Venn analysis (http://bioinformatics.psb.ugent.be/webtools/Venn/) to compare hub genes and DEGs. Further, we selected common genes and used the STRING (https://string‐db.org/) database to construct PPI network and looked for hub genes. Molecular complex detection (MCODE) is a plug‐in to Cytoscape software platform and is used to detect and screen densely interconnect gene modules from the PPI network. The following cut‐off values setting: Degree cut‐off = 4; Node score cut‐off = 0.2; K‐core = 2; Max. depth = 100.

### 2.9 Validation in external data set

We used data set GSE101702 to validate the difference of hub genes between healthy donors and influenza patients with different severity. The gene expression profile was obtained from GEO. The clinical samples of GSE101702 included 107 influenza patients (63 Moderate and 44 Severe) and 52 healthy controls. Then, we compared the difference of hub genes expression between healthy donors and influenza patients with different severity.

### 2.10 Enzyme‐linked immunosorbent assay and clinical validation

Protein was the product of gene expression and the primary executor of biological function. The expression products of 10 hub genes were secretory proteins. We conducted ELISA to measure these expression products of hub genes. The concentration levels of human resistin (RETN), human matrix metalloproteinase‐8 (MMP8), human lipocalin 2 (LCN2), human haptoglobin (HP), human olfactomedin 4 (OLFM4), human neutrophil elastase (ELANE), human bactericidal/permeability‐increasing protein (BPI), human lactoferrin (LTF; Elabscience, Wuhan, China), human transcobalamin I (TCN1) and human defensin α4 (DEFA4) (mlbio, Shanghai, China) in the plasma samples were measured according to manufacturer's instructions, respectively. The clinical samples included 63 influenza patients (34 Moderate and 29 Severe) and 25 healthy controls. We analysed the difference of hub genes expression between healthy donors and influenza patients with different severity. Meanwhile, we compared the difference of hub genes expression between survived and dead cases. Then, we used R packages (‘pROC’ and ‘verification’) to draw ROC curves, calculate p value of hub genes and clinical indicators.

### 2.11 Statistical analysis

Statistical analysis was performed with Graph‐Pad Prism 5.0 and R 3.6.2. And statistical significance was calculated by Student's *t* test, Mann‐Whitney *U* test, chi‐square test or Fisher's exact test, as appropriate. *P < 0.05, **P < 0.01, ***P < 0.001 and ****P < 0.0001 represent significant differences.

## 3 RESULTS

### 3.1 Identification of stable DEGs and function characteristics between influenza samples and healthy donors

**FIGURE 1:**
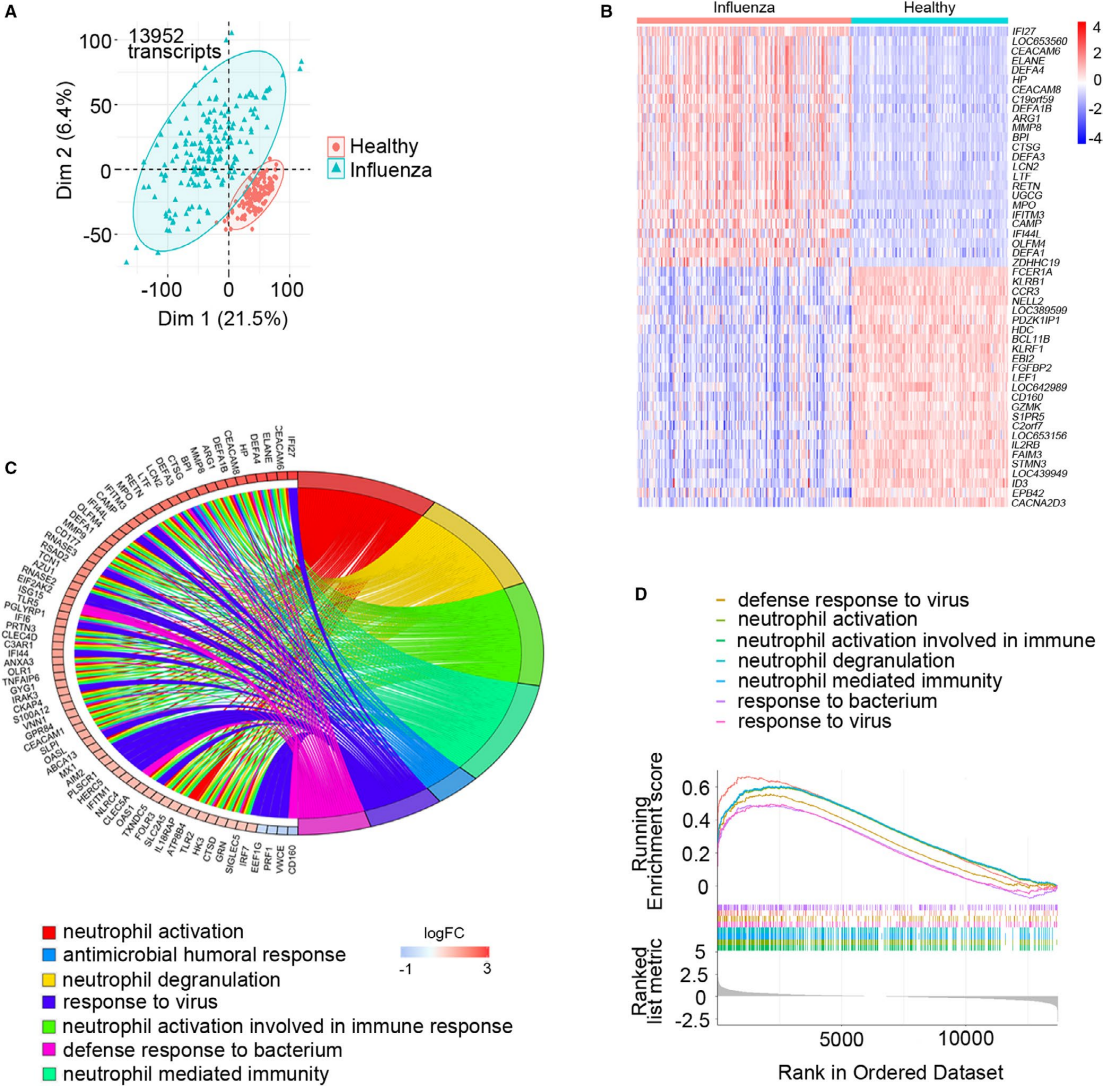
Identification of differentially expressed genes and functional enrichment analyses. A, Principal component analysis was performed with normalized gene expression data, including healthy controls (n = 130) and influenza patients (n = 178) from data set GSE111368. B, Heat map representation of top 50 significant genes, ordered by fold change. Up‐regulated genes are shown in red, and down‐regulated genes are shown in blue. C, Chord plot depicting the relationship between genes and Gene Ontology terms of biological process. D, Gene set enrichment analysis showed seven pathways enriched in influenza patients, and the lines in the lower figure correspond to the genes of each pathway

We applied principal component analysis (PCA) to the 13 952 transcripts among 308 whole‐blood samples. The PCA plot showed that composition of the healthy controls and influenza patient groups were apart from each other, suggesting gene expression profile in the illness group was significantly different from that in the healthy group (Figure 1A). The DEGs between illness and normal people were obtained using R package ‘limma’ analysis method, among which 224 genes were significantly up‐regulated and 133 genes were significantly down‐regulated. The top 25 up‐ and down‐regulated DEGs were shown in the heat map (Figure 1B). The interferon‐stimulated gene (ISG) *IFI27* was extremely elevated and immunoglobulin E receptor α (*FcεRIα*) gene usually decreased. To further characterize the functional status derived from these DEGs, we conducted GO analysis and GSEA across the whole 357 DEGs. These genes were mainly involved in BPs associated with neutrophil activation, neutrophil degranulation, neutrophil activation involved in immune response (Figure 1C). In terms of CCs, the DEGs were mainly enriched in integral components of specific granule and secretory granule lumen (Figure S2A). And the MF terms were glycosaminoglycan binding and lipopolysaccharide binding (Figure S2B). In addition, GSEA results showed that up‐regulated signalling pathways were mainly enriched in neutrophils functions, virus and bacteria‐related immune response in influenza patients (Figure 1D).

### 3.2 Immune landscape associated with characteristics of influenza infection

**FIGURE 2:**
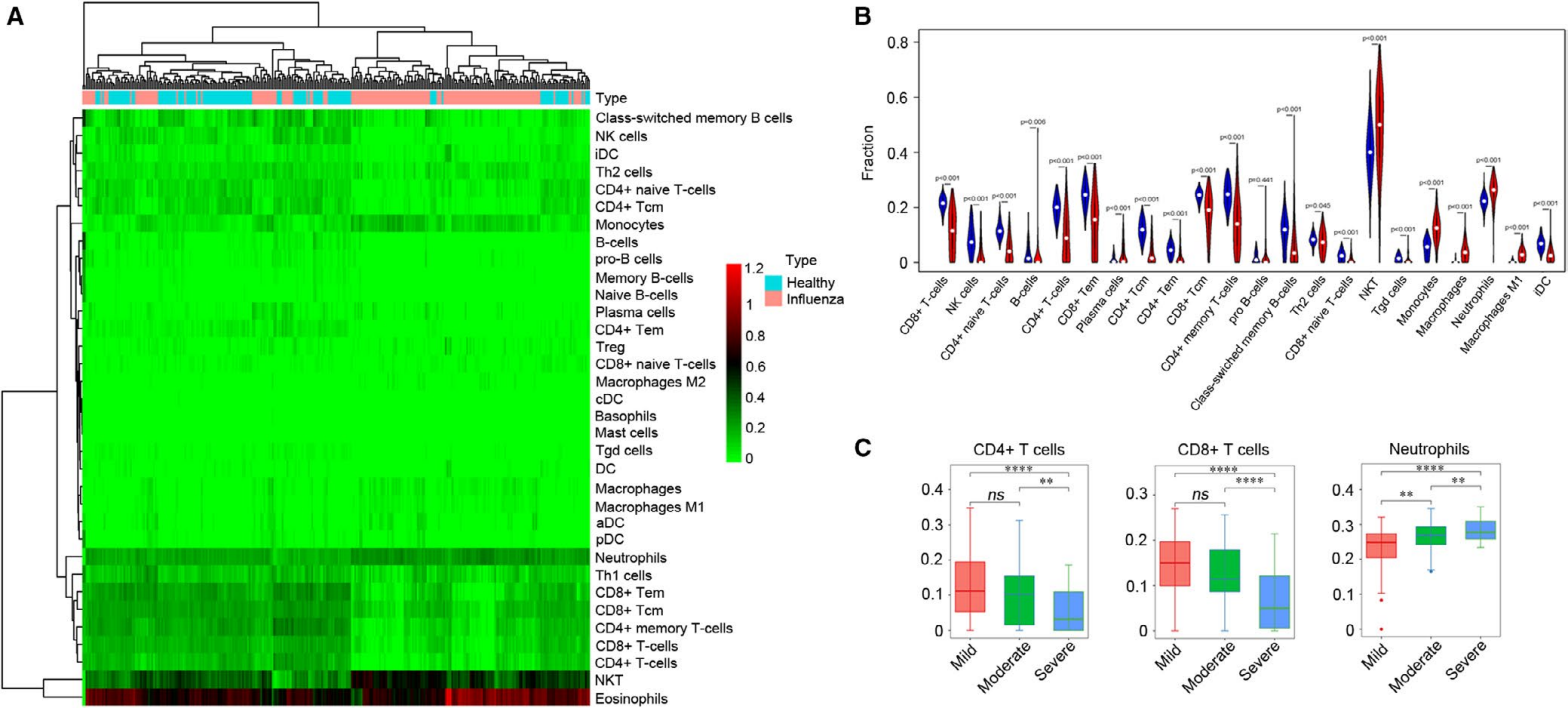
Analysis of immune landscape associated with influenza infection. A, Heat map showing the enrichment score of immune cells between healthy controls and influenza patients from data set GSE111368. The specific enrichment scores of 34 immune cell types were calculated by ssGSEA score‐based method (x‐Cell tool) based on specific gene marker. B, Immune cells abundance in healthy controls and influenza patients. The blue indicates samples of healthy controls, and the red designates the samples of influenza patients. *P* values were obtained using Wilcoxon test. C, Different abundance of Neutrophils, CD4^+^ T cells and CD8^+^ T cells in mild, moderate and severe patients. GSEA, gene set enrichment analysis; ns, not significant. *P < 0.05; **P < 0.01; ***P < 0.001; ****P < 0.0001

Functional enrichment analysis showed that neutrophil function was different between influenza and healthy groups. To explore the distribution characteristics of immune cells in the progression of severe influenza, the microarray data of 308 whole‐blood samples from data set GSE111368 were analysed to evaluate the immune landscape. The specific enrichment scores of 34 immune cell types were calculated by ssGSEA score‐based method (x‐Cell tool) based on specific gene markers between normal controls and influenza samples. The results revealed that neutrophils, monocytes, eosinophils and CD4^+^ T and CD8^+^ T subsets were abundant in the data set, as presented in the corresponding heat map in Figure 2A. Moreover, it was demonstrated that the enrichment score of NKT, monocytes and neutrophils were higher in influenza samples. However, the CD4^+^ T and CD8^+^ T subsets, and B cells were significantly enriched in normal controls compared with influenza samples (Figure 2B). Data from the GSE111368 data set was also used to analyse the enrichment score of immune cell subsets in different severity of influenza infection. The results showed that CD4^+^ T and CD8^+^ T cells significantly decreased in severe influenza patients, but neutrophils increased with disease severity (Figure 2C).

### 3.3 Identification of the key module with WGCNA and its related functions

**FIGURE 3:**
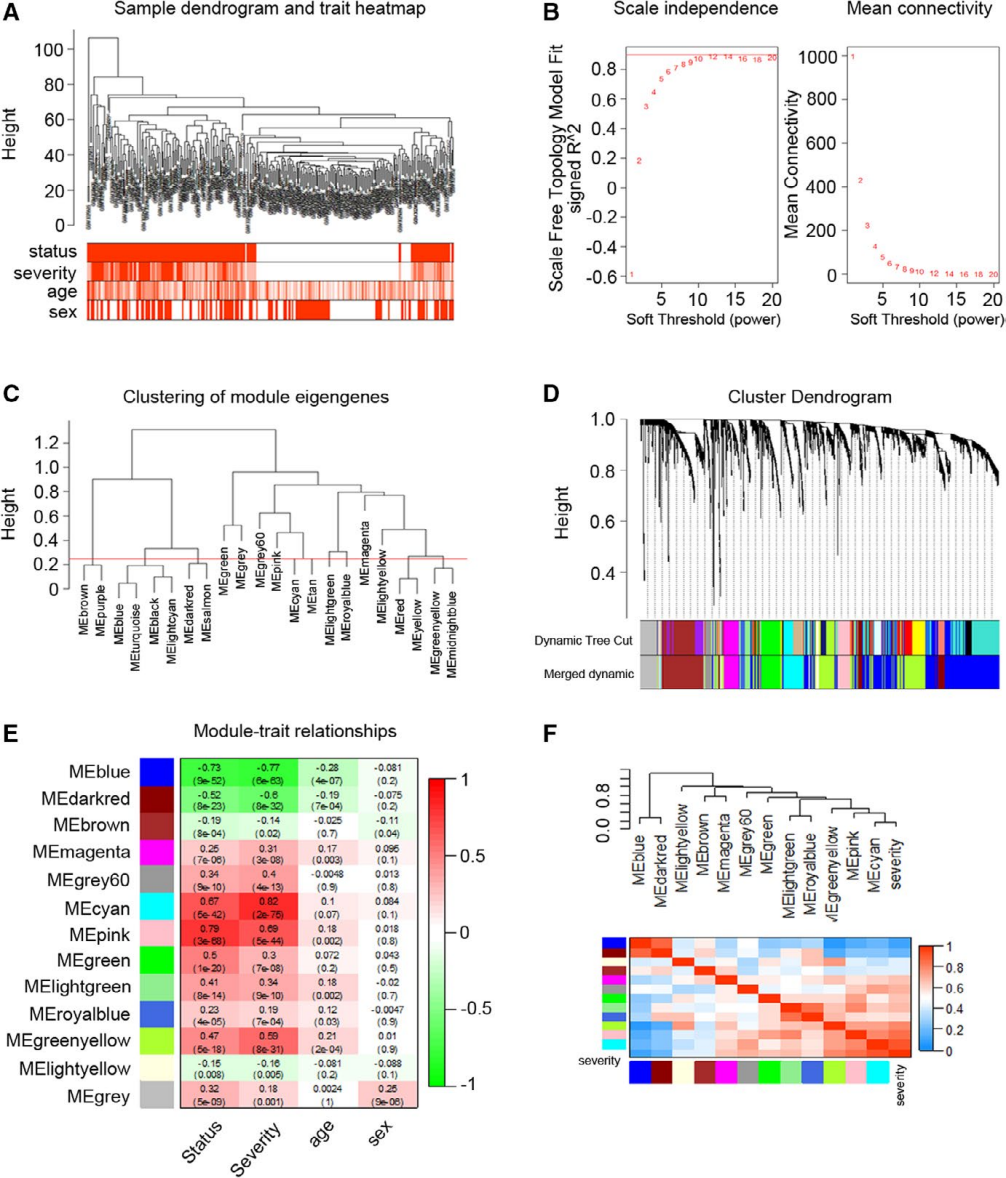
Identification of key modules correlated with disease severity through weighted gene co‐expression network analysis. A, Sample dendrogram and trait heat map (GSE111368). Colour intensity varies positively with age, sex, severity and disease status. B, Analysis of the scale‐free fit index (left) and the mean connectivity (right) for various soft‐thresholding powers. C, Clustering of module eigengenes. The red line indicates cut height (0.25). D, Clustering dendrograms of genes based on a dissimilarity measure (1‐TOM). E, Module‐trait associations were evaluated by correlations between module eigengenes and sample traits. Each cell contains the correlation coefficient and *P* value. A stronger positive correlation was displayed in darker red, and the negative correlation with deeper blue. F, The combination of eigengene dendrogram and heat map indicated that the cyan module is highly related to the severity of influenza infection

To identify the key modules related to severity of influenza infection, data set GSE111368 derived from 308 samples was used to construct the co‐expression network through WGCNA analysis. Clinical traits, including disease status, illness severity, age and sex, were retrieved from raw files (Figure 3A). By setting soft‐thresholding power as 9 (scale‐free *R* ^2^ = 0.85) and cut height as 0.25, we eventually identified 13 modules (Figure 3B‐D). The association between the modules and clinical samples traits was measured by the correlation between module eigengene (ME) values and sample traits and visualized by the heat map profiles. The results showed that the cyan module was the most closely corrected with disease severity (Pearson coefficient = 0.82, *P* = 2E‐75) and was highly related to disease status as well (Pearson coefficient = 0.67, *P* = 5E‐42) (Figure 3E). We plotted dendrogram and heat map to further illustrate the correlated eigengenes, and the dendrogram indicated that the cyan module was significantly related to disease severity (Figure 3F). To elucidate the potential functions of these genes, we conducted GO and KEGG analysis. The enrichment of GO terms in BP, CC and MF, as well as KEGG pathways, were shown in Figure S3A‐B. Function enrichment analysis indicated that genes within the cyan module were mainly involved in neutrophil function and ‘cell cycle’.

### 3.4 Identification of hub genes

**FIGURE 4:**
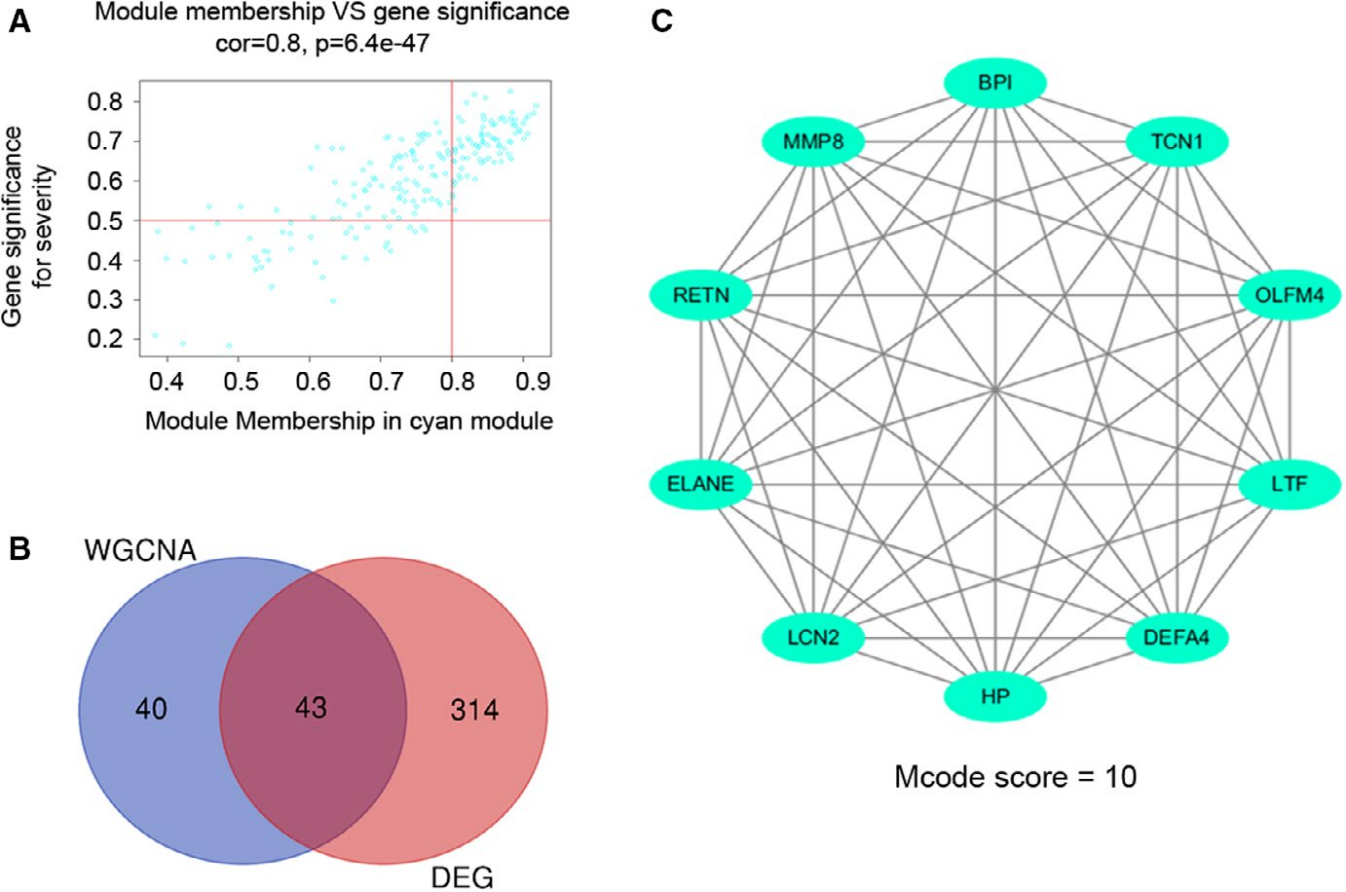
Key hub gene in severe progression of influenza infection. A, A scatter plot of gene significance for influenza severity vs module membership in cyan module (Red line: module membership > 0.8 and gene significance > 0.5 were set to define genes highly correlated with disease severity). B, Venn diagrams indicate overlap of 43 commonly changed genes. Identification of the intersection from the cyan module (WGCNA results, module membership > 0.8 and gene significance > 0.5 were set as the cut‐off criterion) and DEGs (Healthy control vs Influenza infection; Foldchange > 2 and FDR < 0.05 were set as the cut‐off criterion). C, The core module from overlapped genes by PPI (confidence score > 0.9) and Cytoscape software (MCODE: Degree cut‐off = 4; Node score cut‐off = 0.2; K‐core = 2; Max. depth = 100). DEG, differentially expressed gene; FDR, false discovery rate; WGCNA, weighted gene co‐expression network analysis

To screen stable and robust hub genes accurately, 83 key genes with significant correlation both GS and MM were selected by setting MM > 0.8 and GS > 0.5, as shown by the scatter plots (Figure 4A). Furthermore, 43 commonly changed genes shared by cyan module and DEGs were selected (Figure 4B), and these genes had consistently up‐regulated expressed trends. Then, 43 overlapping genes were filtered and constructed into PPI network complex, and the core module was identified with the MCODE score = 10 (Figure 4C). Lastly, these hub genes, including *RETN*, *MMP8*, *LCN2*, *HP*, *OLFM4*, *ELANE*, *TCN1*, *DEFA4*, *BPI* and *LTF*, were selected from core module.

### 3.5 GSEA reveal a close relationship between hub genes and neutrophils functions

**FIGURE 5:**
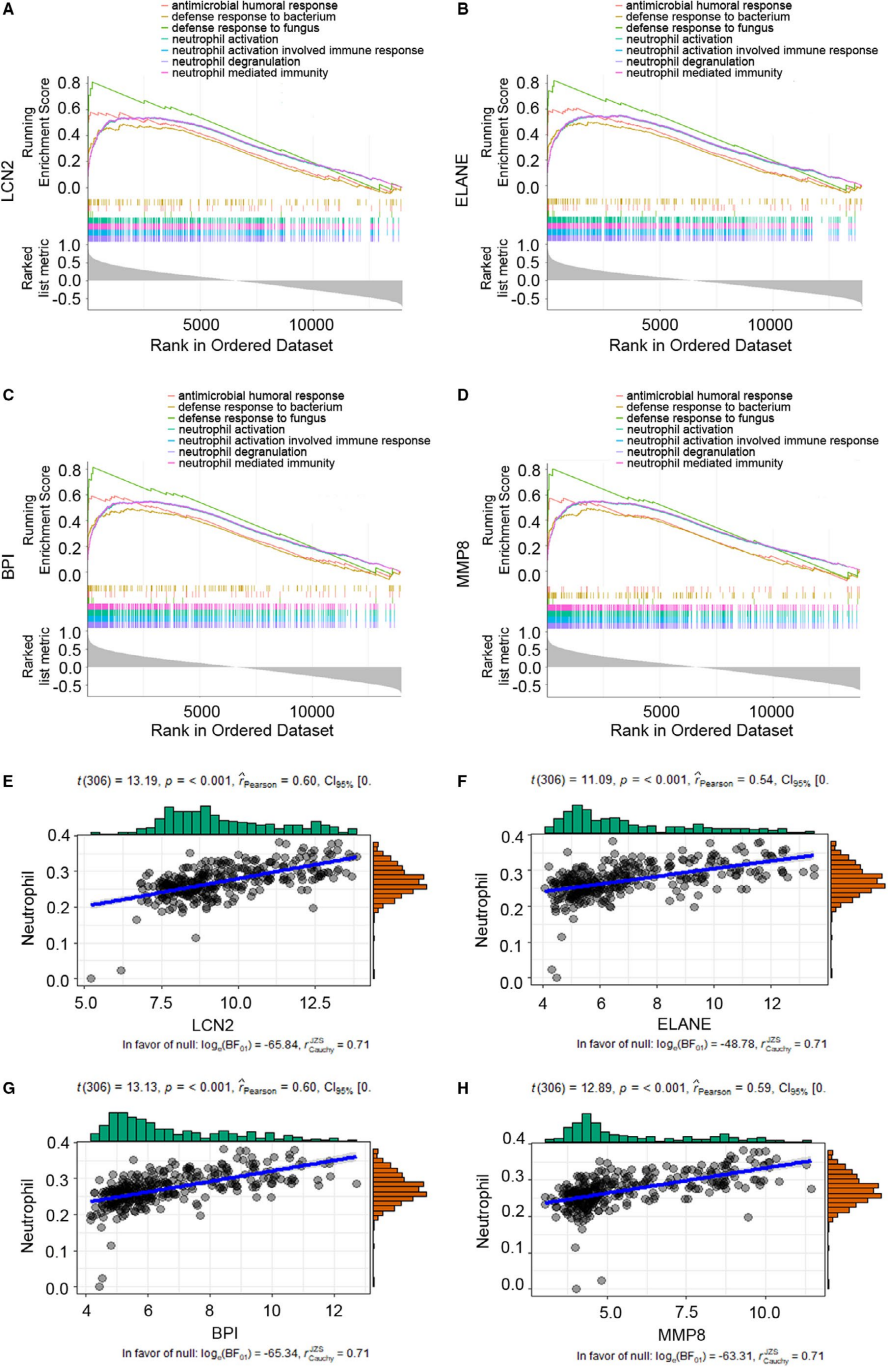
Gene set enrichment analysis (GSEA) of hub gene and association of hub genes' expression with neutrophils. A‐D, seven gene sets enriched in groups with positive correlated with single hub genes (GSE111368). A, *LCN2*; B, *ELANE*; C, *BPI* and D, *MMP8*. E‐H, Association of E, *LCN2*; F, *ELANE*; G, *BPI* and H, *MMP8* genes expression with Neutrophils in influenza infection. Each plot represents a sample

To clarify the potential functions of hub gene, we performed GSEA. As shown in Figure 5A‐D and Figure S4, seven gene sets, including ‘antimicrobial humoral response’, ‘defence response to bacterium’, ‘defence response to fungus’, ‘neutrophil activation’, ‘neutrophil activation involved immune response’, ‘neutrophil degranulation’ and ‘neutrophil mediated immunity’, were enriched in groups with positive correlated with *RETN*, *MMP8*, *LCN2*, *HP*, *OLFM4*, *ELANE*, *TCN1*, *DEFA4*, *BPI* and *LTF*. Overall, these gene sets with higher enrichment scores were all closely associated with neutrophils functions of anti‐bacteria and anti‐fungi.

### 3.6 Neutrophils is associated with hub genes' expression

The result of GSEA showed a close relationship between hub genes and neutrophils function. To further investigate the association between the hub genes expression and neutrophils, we utilized R package ‘ggstatsplot’ to perform correlational analysis between hub genes expression and enrichment score of neutrophils from x‐Cell tool. Interestingly, significant positive associations were observed between these hub genes and enrichment score of neutrophils (Figure 5E‐H; Figure S5). Taken together, all ten hub genes' expression was related to neutrophils.

### 3.7 Validation of candidate genes related to disease severity

**FIGURE 6:**
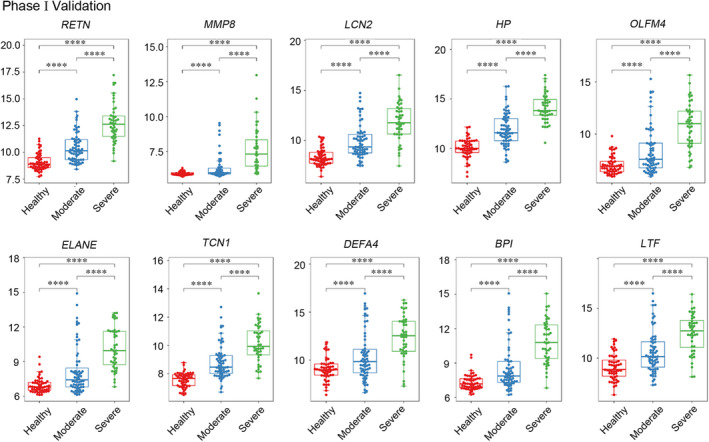
Phase I validation: Candidate genes increased with disease severity. Validation of hub genes in the data set GSE101702. Ten candidate genes highly differentially expressed in severe influenza infection increased with severity of illness. Scatter diagram from the validation set comparing healthy donors (red, n = 52), moderate infection (blue, n = 63) and severe infection (green, n = 44)

To verify the results above, the expression levels of the above ten hub genes were first validated in another data set (GSE101702). The mRNA microarray data set GSE101702 consists of 107 influenza patients and 52 healthy controls. 107 whole‐blood samples were taken from whole enrolled patients (including 63 moderate patients and 44 severe patients). The reconfirmed results showed that expression levels of ten hub genes in influenza patients were higher than normal controls and significantly increased with disease severity (Figure 6).

### 3.8 Candidate gene encoding proteins could evaluate disease severity and predict patient outcome

**FIGURE 7:**
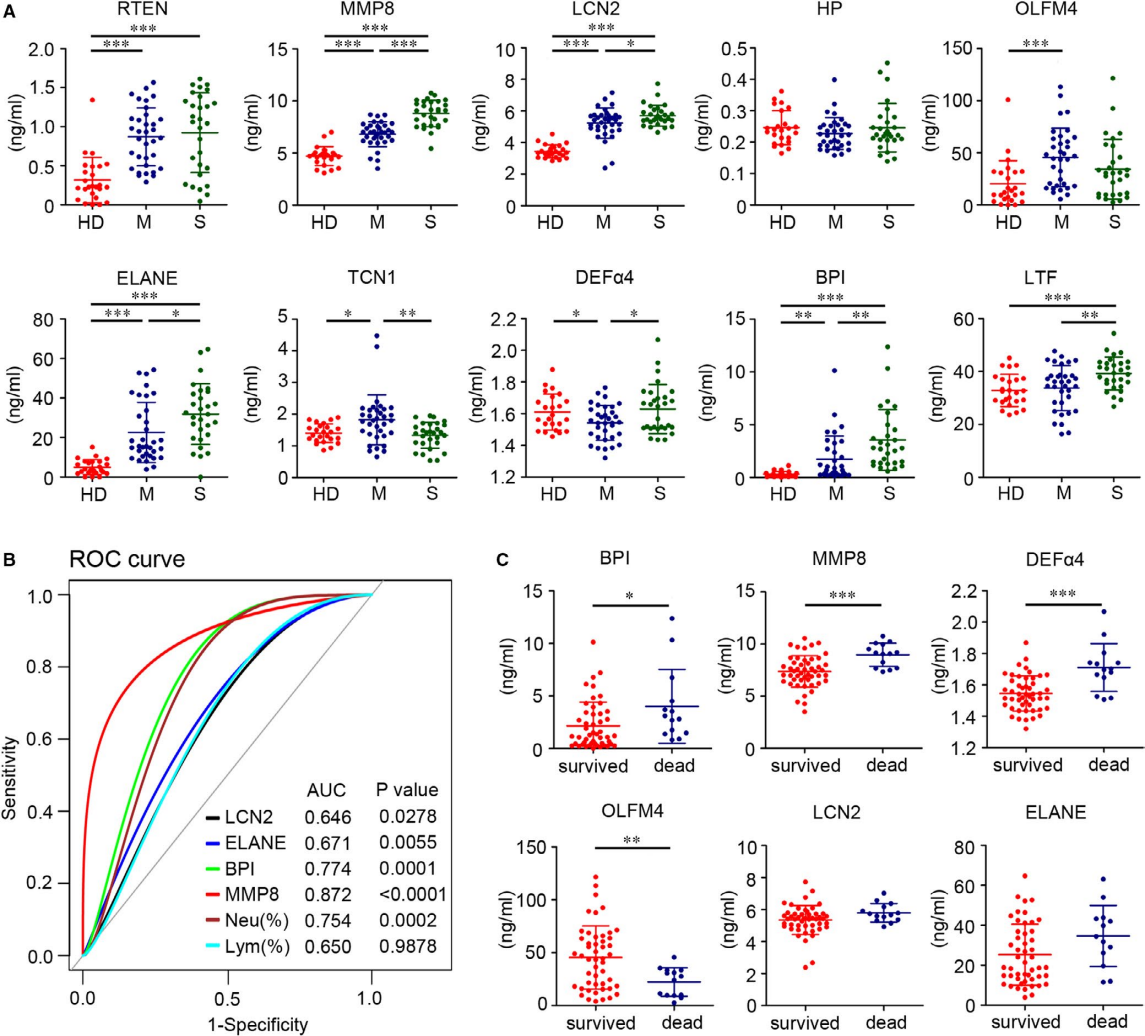
Phase II validation: Candidate proteins evaluate disease severity and predict patient outcome. A, Concentration levels of RETN, MMP8, LCN2, HP, OLFM4, ELANE, TCN1, DEFα4, BPI and LTF in plasma obtained at the first sampling time‐point (T1) from healthy donors (n = 25) and influenza patients (n = 63) and presented as scatter diagram. HD: Healthy donors; M: Moderate patient (n = 34); S: Severe patients (n = 29). B, Severity prediction using LCN2, BPI, ELANE and MMP8 expression levels, and proportion of neutrophils and lymphocytes. Receiver‐operator characteristic (ROC) curves of four candidate proteins and two clinical indicators to predict disease severity: ROC‐AUC (LCN2: 0.646; BPI: 0.774; ELANE: 0.671; MMP8: 0.872; Proportion of neutrophils: 0.754; Proportion of Lymphocytes: 0.650). C, The protein levels of BPI, MMP8, DEFα4, OLFM4, LCN2 and ELANE in survivors (n = 49) and non‐survivors (n = 14) with influenza infection. Statistical significance is determined by unpaired *t* test. *P < 0.05, **P < 0.01, ***P < 0.001

Given the prominent roles displayed by candidate genes in the procession of influenza infection, we suggested that their expression levels could reflect disease status and predict patient outcome. To verify this hypothesis, the protein levels of key candidate gene encoding proteins were detected using ELISA. The concentrations of the RETN, MMP8, LCN2, ELANE and BPI in plasma tended to be higher in influenza patients than healthy donors. In addition, *MMP8*, *LCN2*, *ELANE* and *BPI* encoding proteins increased with disease severity. However, in terms of HP, no or weak differences were observed when plotted against illness severity. Furthermore, concentrations of TCN1 proteins decreased in plasma in patients with relatively severe disease (Figure 7A). Furthermore, receiver operating characteristic (ROC) curves showed their diagnostic value as biomarkers for severe influenza. Area under the curve (AUC) analysis of ROC showed that LCN2, BPI, ELANE and MMP8 expression represented severe influenza infection. In addition, BPI and MMP8 expression better distinguished severe from non‐severe than the proportion of lymphocytes and neutrophils. (BPI: AUC 0.774; MMP8: AUC 0.872) (Figure 7B). To further evaluate whether expression levels of candidate proteins could predict the illness outcome, we performed a comparative analysis to test the levels of candidate proteins in all influenza populations. The results showed that BPI, MMP8 and DEFα4 had a higher expression in dead than survived people, but OLFM4 was the opposite (Figure 7C). Collectively, these findings suggested that some candidate proteins were linked to disease status or illness outcome during influenza infection.

## 4 DISCUSSION

To our knowledge, our study is the first one to apply DEGs analysis combined with WGCNA algorithm to search novel hub genes related to pathogenesis of severe influenza. In addition, our study is also the first to verify these screened key genes in both another new data set and independent clinical plasma samples and to analyse the correlation between hub gene encoding proteins and disease severity or clinical outcome. These findings provide additional insights on understanding the molecular mechanism of severe influenza development.

Numerous studies have been conducted to reveal the causes and immunological mechanisms of progression in severe influenza, by which they could assist in designing treatment strategies. Nonetheless, the morbidity and mortality of influenza infection is still very high in the past several decades. Most studies have identified some host factors associated with severe influenza, however, only focused on a single genetic event (ie genetic susceptibility).^15^, ^16^, ^17^, ^18^, ^19^ Recently, transcriptomics studies also captured extensive gene expression profiles of host response and these results indicated that structure of gene sets and their functions were different across the full range of normal, moderate and severe infection. However, these findings were only generated from a single cohort study and failed to further clinical validation or detailed functional analysis.^8^, ^9^, ^12^, ^20^

A prospective cohort study discovered discernible differences in gene expression pattern between acute and recovery phase from influenza patients.^21^ To minimize variability, we only captured partial transcriptomics information from data set GSE111368. Therefore, we could analyse the characteristics of whole‐blood RNA in the acute phase of influenza infection. Through integrating microarray data from normal people and influenza patients, we identified 357 robust DEGs. Among them, some had greater significance for influenza infection than others, such as *IFI27* (also called *ISG12*) and *IFITM3* (both are up‐regulated genes), have been reported widely and in details.^11^, ^15^, ^22^ Interestingly, both of them belonged to well‐known ISGs.^23^ It was suggested that response to interferon signalling and/or viral infections, as a remarkable indicator, involved in influenza development.

Furthermore, we conducted GO functional enrichment analysis and found that these DEGs were mainly associated with anti‐bacteria response and neutrophil function. These findings mutual confirmed with result of immune infiltration analysis, from which we conclude that neutrophils‐related host response played a pivotal role in influenza infection. Similarly, it has been known that neutrophils, as vital practitioners of microbe‐killing, can defend against bacteria, fungi and protozoa through phagocytosis, degranulation, releasing lytic enzymes and reactive oxygen species.^24^ Aside from these traditional mechanisms, they also perform their killing function by releasing decondensed chromatins and granule proteins, which are collectively called neutrophil extracellular traps (NETs), into the extracellular space.^25^, ^26^, ^27^, ^28^ The biological functions of DEGs closely match the mechanism of biocidal action of NETs. Thus, we can speculate that the activation of neutrophils and production of NETs were the most represented signatures in severe influenza. These results were consistent with previous analysis and reports.^8^, ^12^, ^29^

Weighted gene co‐expression network analysis, as widely used bioinformatics method, focused on the relationship between co‐expression modules and clinical traits at transcriptome level^14^; thus, this approach can provide insight into complementary information related to phenotypic traits. Among 13 modules, the cyan module is the key one involved in influenza severity and its contained genes' pathway enriched in the ‘cell cycle’, which is consistent with previous reports.^8^ It has been reported that ‘cell cycle’ plays a pivotal role in host‐virus interaction. Influenza viruses could escape the host restriction and facilitate their own replication through changing the cell cycle transition points.^30^, ^31^, ^32^ Therefore, these findings provide a possible clinically relevant to better understand the relationship between immune cell proliferation and viral load change.

Ten hub genes were identified using DEGs analysis combined with WGCNA algorithm, and almost all of these genes were neutrophils‐related. Although neutrophils ameliorate lung damage and delay the development of mild flu‐like symptoms to severe or critical clinical illness,^33^ cumulative evidence also suggested that excessive pulmonary infiltration of neutrophils was responsible for adverse outcome of influenza infection.^34^, ^35^, ^36^, ^37^ For example, the prolonged action of NETs might accelerate local damage because of higher immunogenicity.^38^ It has been shown that increased release of NETs was correlated with disease severity and might be a significant guideline to predict the poor prognosis during influenza infection.^39^ However, testing complexity and biased observations limited its clinical application.^40^ Thus, searching alternative indicators might provide more innovative clues to achieve faster and easier detection. NETs are composed of decondensed chromatin fibres coated with antimicrobial proteins, such as histones, neutrophil elastase (ELANE), myeloperoxidase and α‐defensins (DEFα).^25^ Our integrated analysis and validation results also again illustrate that these candidate genes, including *ELANE*, *DEFA4*, involved in NETs formation, could cause or participate in the process of severe influenza.

Neutrophils are essential for the initiation and maintenance of inflammation response as well. Although the neutrophil counts were not statistically significant between moderate and severe patients (*P* = 0.0557; Table 1), closely related genes and their encoding proteins were generally more abundant in relatively severe cases. It also reflected disease status or predicted influenza patients' outcome. Among them, some proteins related to specific neutrophils granules, such as bactericidal/permeability‐increasing (BPI) protein and matrix metalloproteinase‐8 (MMP8) protein, were generally more abundant in relative severe influenza cases.^41^, ^42^ Specially, it has been reported recently that originally identified as neutrophil collagenase, MMP8 was elevated after influenza infection.^43^ BPI, usually as antimicrobial proteins and peptides, could also destroy the viral particles completely to inhibit infectious abilities of influenza viruses.^44^ In this study, we found that MMP8 and BPI were sensitive to disease status evaluation, which suggested that it could provide potential clinical application values as candidate biomarkers in the future. Resistin (RETN) could induce myeloid/granulocyte specific protein expression such as lipocalin 2 (LCN2) and lactoferrin (LTF), which promote formation of mature neutrophils.^45^ Indeed, ROC curves showed that LCN2 could serve as promising biomarkers to define and distinguish severe influenza with high sensitivity and accuracy. In terms of mutual functions, LTF and LCN2, as iron‐associated glycoprotein, exhibit anti‐inflammatory and anti‐bacterial properties.^46^, ^47^ However, RETN has recently reported to directly inhibit bacterial killing in neutrophils.^48^ In addition, Transcobalamin I (TCN1) encoding proteins in plasma tended to be lower in severe patients than in moderate patients. TCN1, as a vitamin B_12_ (VitB_12_) binding protein, respectively, expressed in mature neutrophils, and most strongly at the stages of myeloid development and granulocyte differentiation.^49^ As of yet, the mechanisms to explain decreased protein levels of TCN1 in severe influenza infection are unknown, and the phenomenon and causes deserve further investigation.

In terms of the gene and protein expression in the validation II and I, they didn't exactly match. The possible reasons for the results mentioned above lie in the following aspects. According to the central dogma, the main links of gene expression include transcription and protein synthesis, but there existed complex post‐transcriptional and post‐translational regulation, such as epigenetic modification.^50^ It might cause inconsistent trends of gene expression between transcriptional and protein levels. In addition, the difference in detection time might be a reason that cannot be ignored. For example, when mRNA peaks, protein levels were still changing.^51^ Last but not least, some specific secreted proteins expressed differently because of different sources from tissues and organs or different disease severity. For example, TCN1, as a VitB_12_ binding protein, may be more related to gastrointestinal illness than just severe influenza infection.^49^

We wanted to point out that Jake's research generated from British influenza patients and their further analysis results mainly emphasized the patterns change in progression of severe influenza.^9^ However, enrolled patients in Benjamin's study came from different countries (including Australia, Canada and Germany) and available network analysis in this study revealed different disease modules were associated with infection severity.^8^ Although these two studies used different platforms and population for gene expression analysis, key hub genes and their expression trends essentially agree with each other. The reason may be that the enrolled population criteria were similar and the analysis methods and obtained data were normalized or standardized. Based on these findings, we proposed that the expression of these hub genes is universal in severe influenza population. Of course, their expression and related function in influenza infection also need to be elucidated in the future.

Some limitations in the study have to be acknowledged. A significant number of DEGs were associated with anti‐bacterial function of neutrophils, including neutrophils degranulation, NETs formation and so on. However, it remains unclear whether modulation of these host factors was specific for severe influenza infection. Hence, the main problem should be clarified through further study, such as controlled animal experiments and even randomized controlled clinical trials in humans.

In conclusion, our comprehensive bioinformatics analysis results showed that neutrophils activation and increase of anti‐bacterial activity were sets of typical characteristics of severe influenza. Several hub genes related to neutrophil activation and anti‐bacterial humoral response were more highly associated with severe disease. New data sets and clinical samples validation revealed *BPI*, MMP8 and their encoding proteins, as indicators of high sensitivity and specificity, could reflect disease severity and predict outcome. Overall, these findings could significantly improve our understanding of causes and underlying biological events in severe influenza infection. And it could also provide new insights into molecular mechanism research and clinical diagnosis or treatment of severe influenza.

## CONFLICT OF INTEREST

The authors declare that the research was conducted in the absence of any commercial or financial relationships that could be construed as a potential conflict of interest.

## AUTHOR CONTRIBUTIONS

**Shuai Liu:** Data curation (equal); Formal analysis (equal); Investigation (equal); Methodology (equal); Resources (equal); Writing‐original draft (equal). **Zhisheng Huang:** Conceptualization (equal); Data curation (equal); Writing‐original draft (equal). **Xiaoyan Deng:** Investigation (equal); Validation (equal). **Xiaohui Zou:** Data curation (equal); Formal analysis (equal); Supervision (equal); Validation (equal). **Hui Li:** Formal analysis (equal); Investigation (equal); Methodology (equal); Validation (equal); Visualization (equal). **Shengrui Mu:** Conceptualization (equal); Data curation (equal); Resources (equal); Software (equal); Supervision (equal). **Bin Cao:** Funding acquisition (lead); Project administration (lead).

## Supporting information

Supplementary Material

## Data Availability

Publicly available data sets were analysed in this study. These data can be found in GSE111368 (https://www.ncbi.nlm.nih.gov/geo/query/acc.cgi?acc=GSE111368) and GSE101702 (https://www.ncbi.nlm.nih.gov/geo/query/acc.cgi?acc=GSE111368).

## References

[1] Li L , Liu Y , Wu P , et al. Influenza‐associated excess respiratory mortality in China, 2010–15: a population‐based study. Lancet Public Health. 2019;4(9):e473‐e481. 31493844 10.1016/S2468-2667(19)30163-X PMC8736690

[2] Wu S , Wei Z , Greene CM , et al. Mortality burden from seasonal influenza and 2009 H1N1 pandemic influenza in Beijing, China, 2007–2013. Influenza Other Respir Viruses. 2018;12(1):88‐97. 29054110 10.1111/irv.12515 PMC5818349

[3] Zhao Y , Zhang Y‐H , Denney L , et al. High levels of virus‐specific CD4^+^ T cells predict severe pandemic influenza A virus infection. Am J Respir Crit Care Med. 2012;186(12):1292‐1297. 23087026 10.1164/rccm.201207-1245OC

[4] Shi SJ , Li H , Liu M , et al. Mortality prediction to hospitalized patients with influenza pneumonia: PO(2)/FiO(2) combined lymphocyte count is the answer. Clin Respir J. 2017;11(3):352‐360. 26148709 10.1111/crj.12346 PMC7162301

[5] Wang Z , Wan Y , Qiu C , et al. Recovery from severe H7N9 disease is associated with diverse response mechanisms dominated by CD8^+^ T cells. Nat Commun. 2015;6:6833. 25967273 10.1038/ncomms7833 PMC4479016

[6] Ingram PR , Inglis T , Moxon D , Speers D . Procalcitonin and C‐reactive protein in severe 2009 H1N1 influenza infection. Intensive Care Med. 2010;36(3):528‐532. 20069274 10.1007/s00134-009-1746-3 PMC7080172

[7] Duarte PA , Bredt CS , Bredt Jr GL , et al. Procalcitonin in patients with influenza A (H1N1) infection and acute respiratory failure. Einstein (Sao Paulo, Brazil). 2011;9(1):52‐55. 10.1590/S1679-45082011AO1878 26760553

[8] Tang BM , Shojaei M , Teoh S , et al. Neutrophils‐related host factors associated with severe disease and fatality in patients with influenza infection. Nat Commun. 2019;10(1):3422. 31366921 10.1038/s41467-019-11249-y PMC6668409

[9] Dunning J , Blankley S , Hoang LT , et al. Progression of whole‐blood transcriptional signatures from interferon‐induced to neutrophil‐associated patterns in severe influenza. Nat Immunol. 2018;19(6):625‐635. 29777224 10.1038/s41590-018-0111-5 PMC5985949

[10] Parnell GP , McLean AS , Booth DR , et al. A distinct influenza infection signature in the blood transcriptome of patients with severe community‐acquired pneumonia. Critical Care (London, England). 2012;16(4):R157. 10.1186/cc11477 PMC3580747 22898401

[11] Tang BM , Shojaei M , Parnell GP , et al. A novel immune biomarker IFI27 discriminates between influenza and bacteria in patients with suspected respiratory infection. Eur Respir J. 2017;49(6). 10.1183/13993003.02098-2016 28619954

[12] Zerbib Y , Jenkins EK , Shojaei M , et al. Pathway mapping of leukocyte transcriptome in influenza patients reveals distinct pathogenic mechanisms associated with progression to severe infection. BMC Med Genomics. 2020;13(1):28. 32066441 10.1186/s12920-020-0672-7 PMC7027223

[13] Yu G , Wang LG , Han Y , He QY . clusterProfiler: an R package for comparing biological themes among gene clusters. OMICS. 2012;16(5):284‐287. 22455463 10.1089/omi.2011.0118 PMC3339379

[14] Langfelder P , Horvath S . WGCNA: an R package for weighted correlation network analysis. BMC Bioinformatics. 2008;9:559. 19114008 10.1186/1471-2105-9-559 PMC2631488

[15] Allen EK , Randolph AG , Bhangale T , et al. SNP‐mediated disruption of CTCF binding at the IFITM3 promoter is associated with risk of severe influenza in humans. Nat Med. 2017;23(8):975‐983. 28714988 10.1038/nm.4370 PMC5702558

[16] Ciancanelli MJ , Abel L , Zhang SY , Casanova JL . Host genetics of severe influenza: from mouse Mx1 to human IRF7. Curr Opin Immunol. 2016;38:109‐120. 26761402 10.1016/j.coi.2015.12.002 PMC4733643

[17] Ciancanelli MJ , Huang SX , Luthra P , et al. Infectious disease. Life‐threatening influenza and impaired interferon amplification in human IRF7 deficiency. Science (New York, NY). 2015;348(6233):448‐453. 10.1126/science.aaa1578 PMC4431581 25814066

[18] Hernandez N , Melki I , Jing H , et al. Life‐threatening influenza pneumonitis in a child with inherited IRF9 deficiency. J Exp Med. 2018;215(10):2567‐2585. 30143481 10.1084/jem.20180628 PMC6170168

[19] Wang Z , Zhang A , Wan Y , et al. Early hypercytokinemia is associated with interferon‐induced transmembrane protein‐3 dysfunction and predictive of fatal H7N9 infection. Proc Natl Acad Sci USA. 2014;111(2):769‐774. 24367104 10.1073/pnas.1321748111 PMC3896201

[20] Ramilo O , Allman W , Chung W , et al. Gene expression patterns in blood leukocytes discriminate patients with acute infections. Blood. 2007;109(5):2066‐2077. 17105821 10.1182/blood-2006-02-002477 PMC1801073

[21] Zhai Y , Franco LM , Atmar RL , et al. Host transcriptional response to influenza and other acute respiratory viral infections—a prospective cohort study. PLoS Pathog. 2015;11(6):e1004869. 26070066 10.1371/journal.ppat.1004869 PMC4466531

[22] Desai TM , Marin M , Chin CR , Savidis G , Brass AL , Melikyan GB . IFITM3 restricts influenza A virus entry by blocking the formation of fusion pores following virus‐endosome hemifusion. PLoS Pathog. 2014;10(4):e1004048. 24699674 10.1371/journal.ppat.1004048 PMC3974867

[23] Villalón‐Letelier F , Brooks AG , Saunders PM , Londrigan SL , Reading PC . Host cell restriction factors that limit influenza A infection. Viruses. 2017;9(12). 10.3390/v9120376 PMC5744151 29215570

[24] Segal AW . How neutrophils kill microbes. Annu Rev Immunol. 2005;23:197‐223. 15771570 10.1146/annurev.immunol.23.021704.115653 PMC2092448

[25] Brinkmann V , Reichard U , Goosmann C , et al. Neutrophil extracellular traps kill bacteria. Science (New York, NY). 2004;303(5663):1532‐1535. 10.1126/science.1092385 15001782

[26] Urban CF , Reichard U , Brinkmann V , Zychlinsky A . Neutrophil extracellular traps capture and kill *Candida albicans* yeast and hyphal forms. Cell Microbiol. 2006;8(4):668‐676. 16548892 10.1111/j.1462-5822.2005.00659.x

[27] Guimarães‐Costa AB , Nascimento MT , Froment GS , et al. *Leishmania amazonensis* promastigotes induce and are killed by neutrophil extracellular traps. Proc Natl Acad Sci USA. 2009;106(16):6748‐6753. 19346483 10.1073/pnas.0900226106 PMC2672475

[28] McCormick A , Heesemann L , Wagener J , et al. NETs formed by human neutrophils inhibit growth of the pathogenic mold *Aspergillus fumigatus* . Microbes Infect. 2010;12(12–13):928‐936. 20603224 10.1016/j.micinf.2010.06.009

[29] Narasaraju T , Yang E , Samy RP , et al. Excessive neutrophils and neutrophil extracellular traps contribute to acute lung injury of influenza pneumonitis. Am J Pathol. 2011;179(1):199‐210. 21703402 10.1016/j.ajpath.2011.03.013 PMC3123873

[30] Jiang W , Wang Q , Chen S , et al. Influenza A virus NS1 induces G0/G1 cell cycle arrest by inhibiting the expression and activity of RhoA protein. J Virol. 2013;87(6):3039‐3052. 23283961 10.1128/JVI.03176-12 PMC3592114

[31] He Y , Xu KE , Keiner B , et al. Influenza A virus replication induces cell cycle arrest in G0/G1 phase. J Virol. 2010;84(24):12832‐12840. 20861262 10.1128/JVI.01216-10 PMC3004346

[32] Fan Y , Mok C‐P , Chan MCW , et al. Cell cycle‐independent role of cyclin D3 in host restriction of influenza virus infection. J Biol Chem. 2017;292(12):5070‐5088. 28130444 10.1074/jbc.M117.776112 PMC5377818

[33] Tate MD , Deng YM , Jones JE , Anderson GP , Brooks AG , Reading PC . Neutrophils ameliorate lung injury and the development of severe disease during influenza infection. J Immunol (Baltimore, Md: 1950). 2009;183(11):7441‐7450. 10.4049/jimmunol.0902497 19917678

[34] Marcelin G , Aldridge JR , Duan S , et al. Fatal outcome of pandemic H1N1 2009 influenza virus infection is associated with immunopathology and impaired lung repair, not enhanced viral burden, in pregnant mice. J Virol. 2011;85(21):11208‐11219. 21865394 10.1128/JVI.00654-11 PMC3194964

[35] Pechous RD . With friends like these: the complex role of neutrophils in the progression of severe pneumonia. Front Cell Infect Microbiol. 2017;7:160. 28507954 10.3389/fcimb.2017.00160 PMC5410563

[36] Damjanovic D , Small CL , Jeyanathan M , McCormick S , Xing Z . Immunopathology in influenza virus infection: uncoupling the friend from foe. Clin Immunol (Orlando, Fla). 2012;144(1):57‐69. 10.1016/j.clim.2012.05.005 22673491

[37] Mauad T , Hajjar LA , Callegari GD , et al. Lung pathology in fatal novel human influenza A (H1N1) infection. Am J Respir Crit Care Med. 2010;181(1):72‐79. 19875682 10.1164/rccm.200909-1420OC

[38] Cheng OZ , Palaniyar N . NET balancing: a problem in inflammatory lung diseases. Front Immunol. 2013;4:1. 23355837 10.3389/fimmu.2013.00001 PMC3553399

[39] Zhu L , Liu LU , Zhang Y , et al. High level of neutrophil extracellular traps correlates with poor prognosis of severe influenza A infection. J Infect Dis. 2018;217(3):428‐437. 29325098 10.1093/infdis/jix475

[40] Volker B , Christian G , Kühn LI , Arturo Z . Automatic quantification of in vitro NET formation. Front Immunol. 2013;3:413. 23316198 10.3389/fimmu.2012.00413 PMC3540390

[41] Bülow S , Zeller L , Werner M , et al. Bactericidal/permeability‐increasing protein is an enhancer of bacterial lipoprotein recognition. Front Immunol. 2018;9:2768. 30581431 10.3389/fimmu.2018.02768 PMC6293271

[42] Cox JH , Starr AE , Kappelhoff R , Yan R , Roberts CR , Overall CM . Matrix metalloproteinase 8 deficiency in mice exacerbates inflammatory arthritis through delayed neutrophil apoptosis and reduced caspase 11 expression. Arthritis Rheum. 2010;62(12):3645‐3655. 21120997 10.1002/art.27757

[43] Talmi‐Frank D , Altboum Z , Solomonov I , et al. Extracellular matrix proteolysis by MT1‐MMP contributes to influenza‐related tissue damage and mortality. Cell Host Microbe. 2016;20(4):458‐470. 27736644 10.1016/j.chom.2016.09.005

[44] Pinkenburg O , Meyer T , Bannert N , et al. The human antimicrobial protein bactericidal/permeability‐increasing protein (BPI) inhibits the infectivity of influenza A virus. PLoS One. 2016;11(6):e0156929. 27273104 10.1371/journal.pone.0156929 PMC4894568

[45] Patel L , Buckels AC , Kinghorn IJ , et al. Resistin is expressed in human macrophages and directly regulated by PPAR gamma activators. Biochem Biophys Res Comm. 2003;300(2):472‐476. 12504108 10.1016/s0006-291x(02)02841-3

[46] Okubo K , Kamiya M , Urano Y , et al. Lactoferrin suppresses neutrophil extracellular traps release in inflammation. EBioMedicine. 2016;10:204‐215. 27453322 10.1016/j.ebiom.2016.07.012 PMC5006695

[47] Xiao X , Yeoh BS , Vijay‐Kumar M . Lipocalin 2: an emerging player in iron homeostasis and inflammation. Annu Rev Nutr. 2017;37:103‐130. 28628361 10.1146/annurev-nutr-071816-064559

[48] Miller L , Singbartl K , Chroneos ZC , Ruiz‐Velasco V , Lang CH , Bonavia A . Resistin directly inhibits bacterial killing in neutrophils. Intensive Care Med Exp. 2019;7(1):30. 31147868 10.1186/s40635-019-0257-y PMC6542889

[49] Andrès E , Serraj K , Zhu J , Vermorken AJ . The pathophysiology of elevated vitamin B12 in clinical practice. QJM. 2013;106(6):505‐515. 23447660 10.1093/qjmed/hct051

[50] Vogel C , Marcotte EM . Insights into the regulation of protein abundance from proteomic and transcriptomic analyses. Nat Rev Genet. 2012;13(4):227‐232. 22411467 10.1038/nrg3185 PMC3654667

[51] Liu Y , Beyer A , Aebersold R . On the dependency of cellular protein levels on mRNA abundance. Cell. 2016;165(3):535‐550. 27104977 10.1016/j.cell.2016.03.014

