# Supplementary material for: Identification of key candidate biomarkers for severe influenza infection by integrated bioinformatical analysis and initial clinical validation
Source: J Cell Mol Med. 2021 Jan 14;25(3):1725–38. doi: 10.1111/jcmm.16275 (PMC7875920; doi:10.1111/jcmm.16275)
Supplement: Supplementary file 1 — Supplementary Material [file JCMM-25-1725-s001.pdf]

Identification of key candidate biomarkers for severe influenza infection by integrated bioinformatical analysis and initial clinical validation

Supplemental Materials

Figure S1.

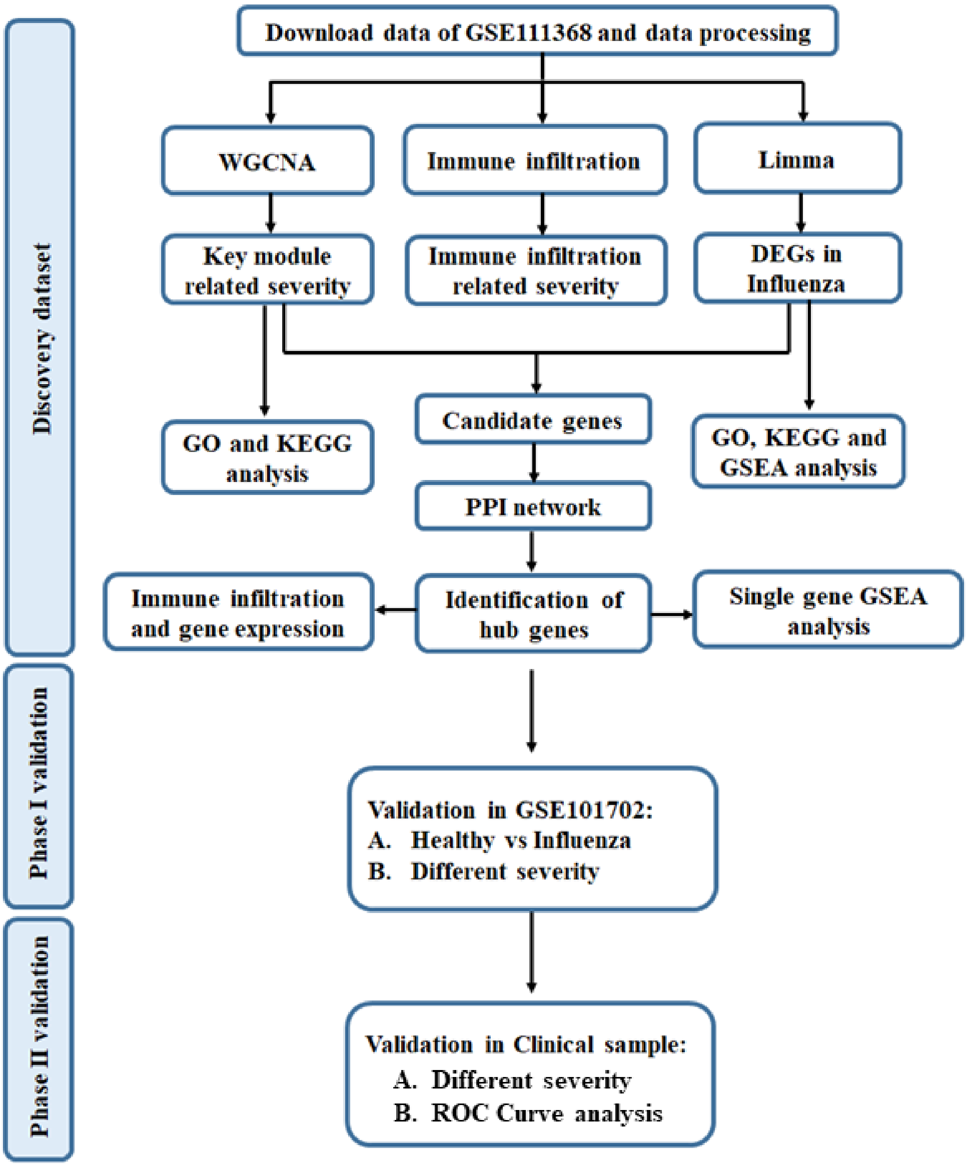

Figure S1. The workflow of the study.

Figure S2.

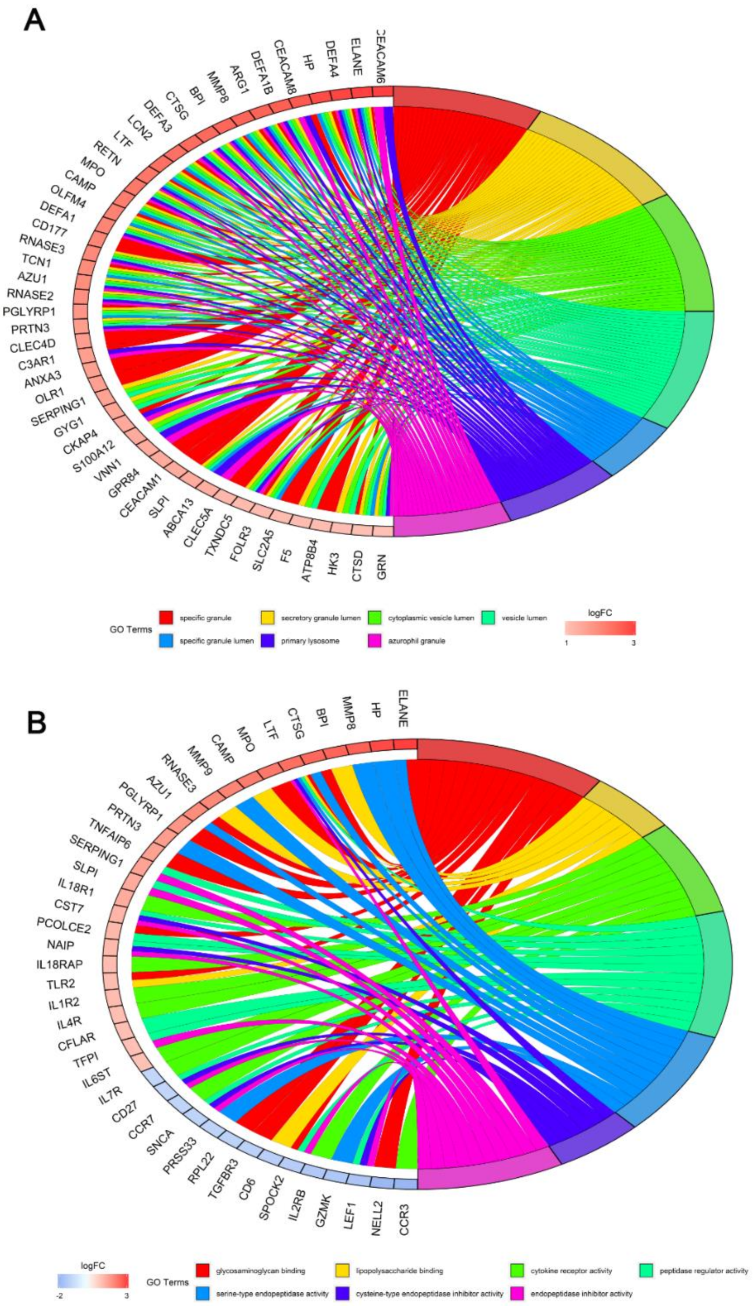

Figure S2. GO analysis of 357 DEGs. A, Chord plot depicting the relationship between genes and Gene ontology (GO) terms of cellular component (CC). B, Chord plot depicting the relationship between genes and GO terms of molecular function (MF).

Figure S3.

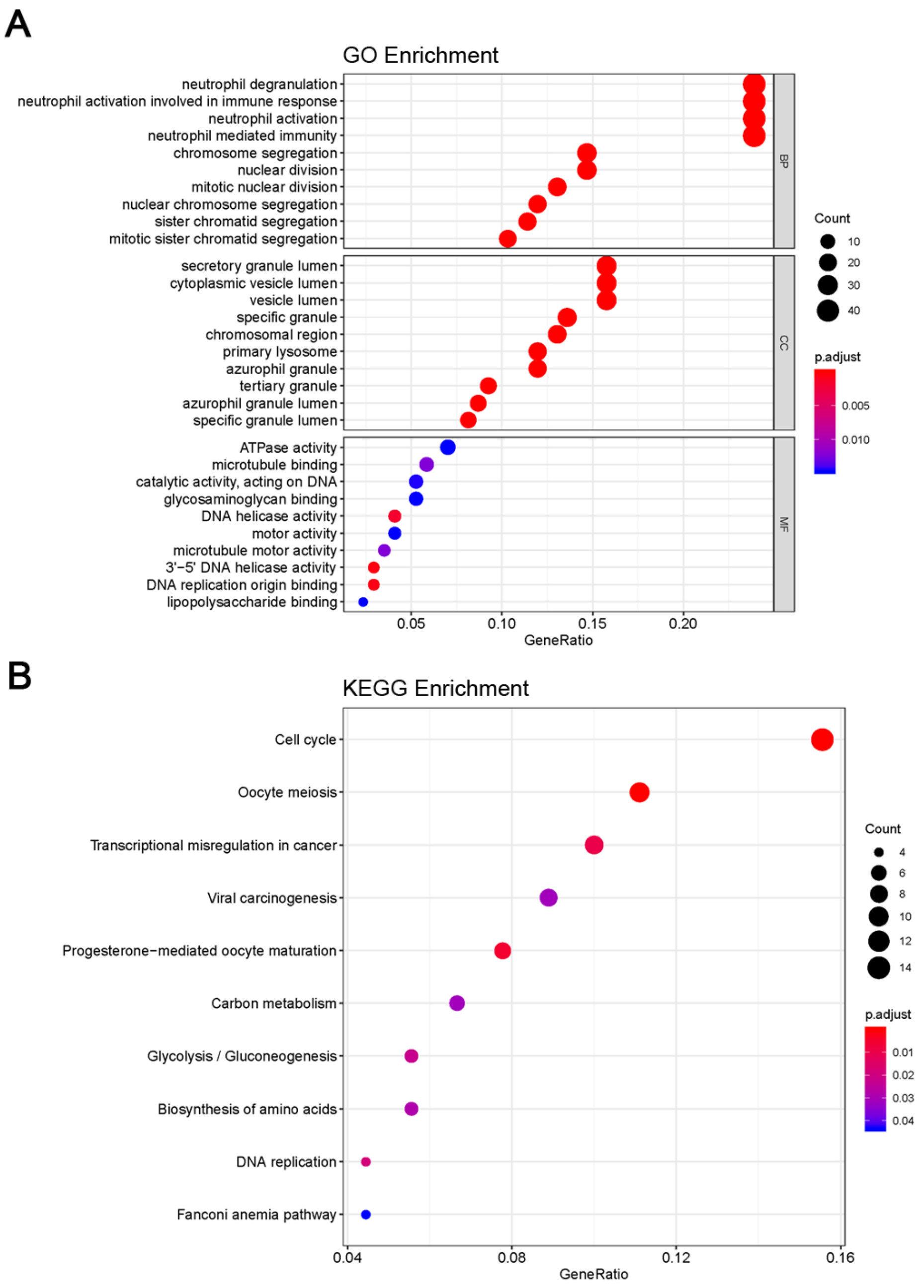

Figure S3. Bubble plot for statistics of GO and KEGG enrichment. A, GO analysis of genes from cyan module. B, KEGG analysis of genes from cyan module. The color and size of the dots represent the range of the p-value and the number of genes mapped to the indicated GO or KEGG terms. Gene-Ratio is defined as the ratio of the differentially expressed gene number to the total gene number.

Figure S4.

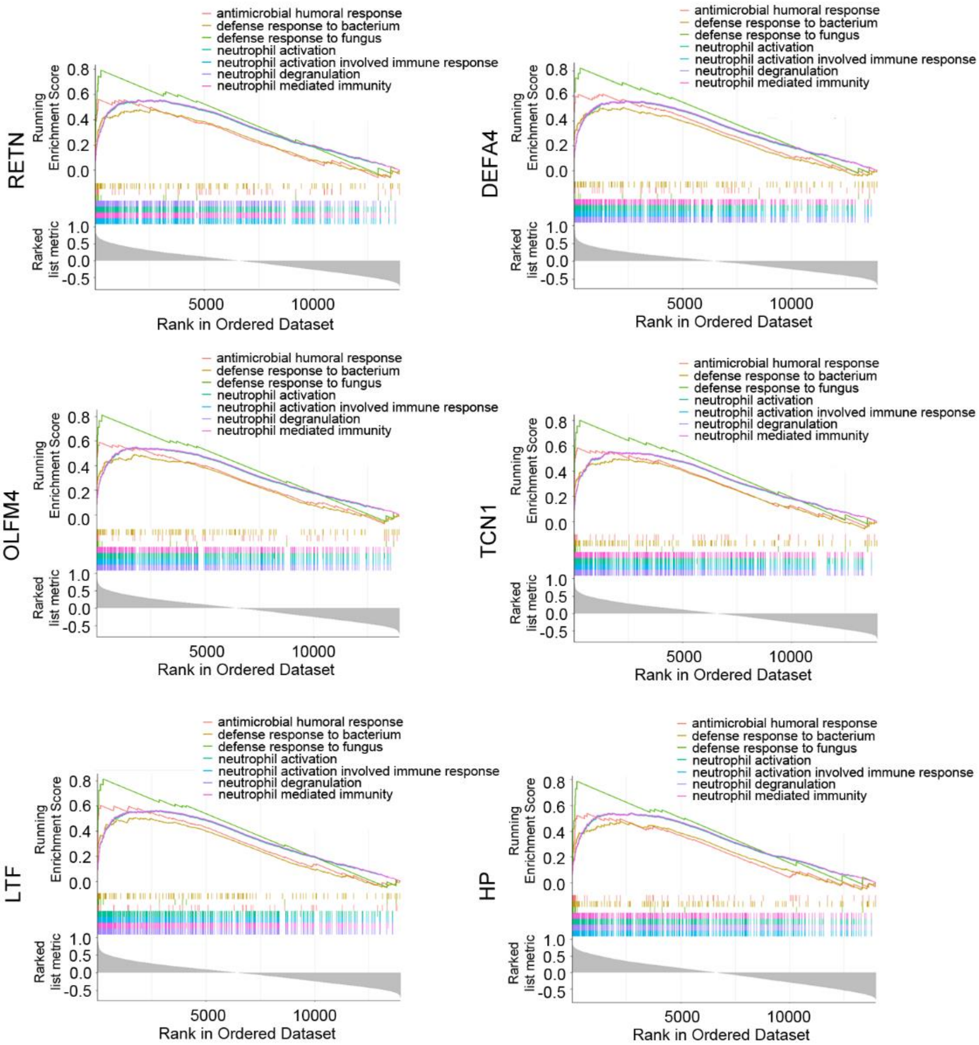

Figure S4. Seven gene sets enriched in groups with positive correlated with single hub genes.

Figure S5.

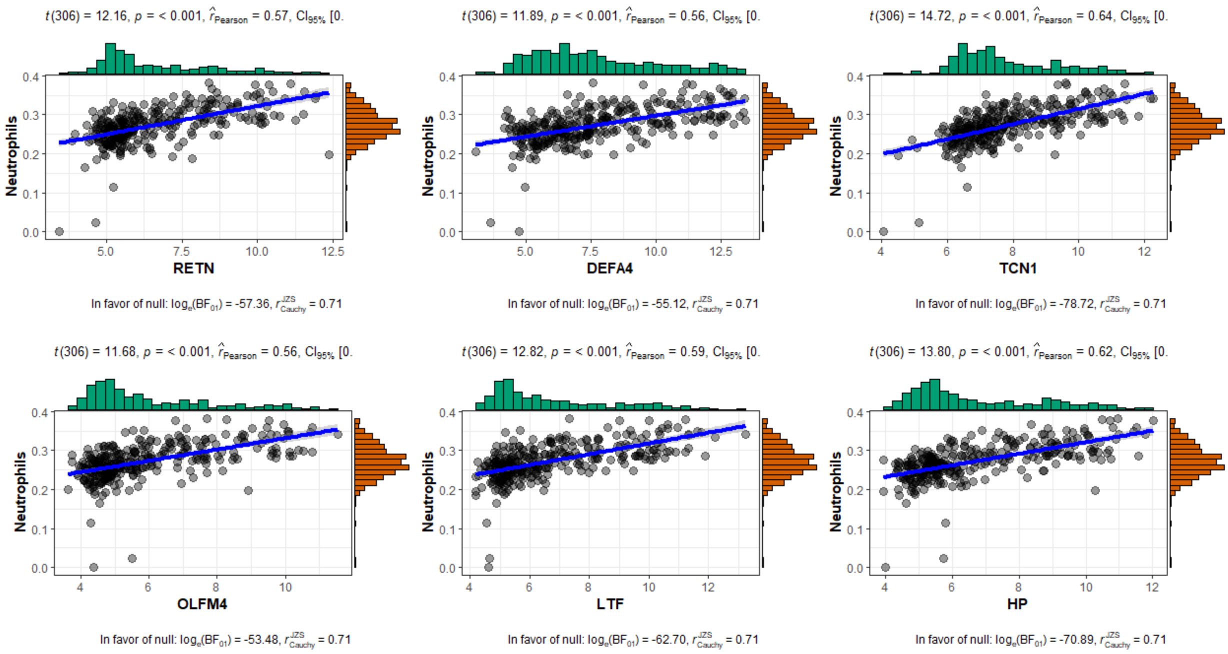

Figure S5. Association of hub genes expression with neutrophils in influenza infection. Each plot represents a sample.

Figure S6.

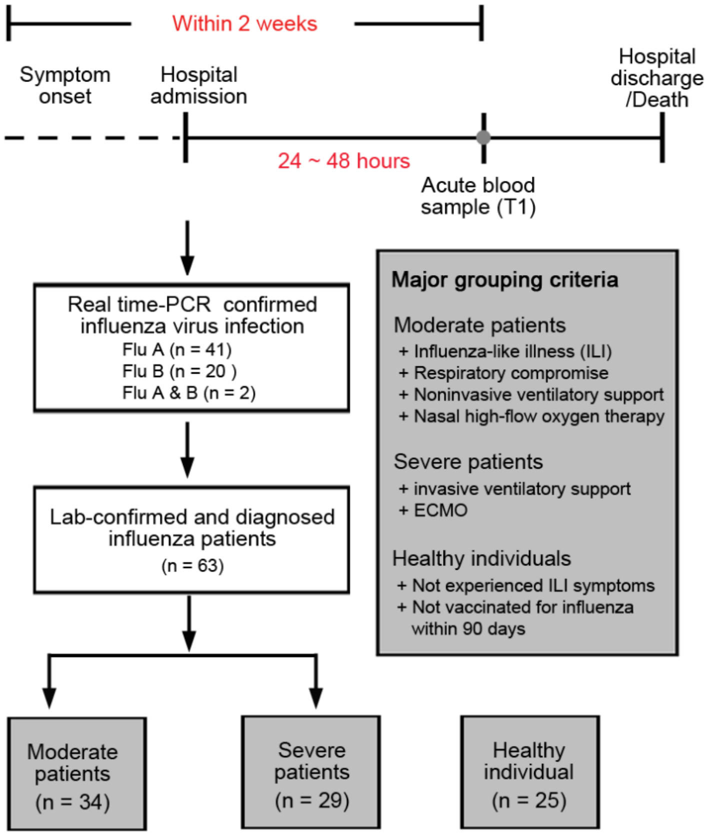

Figure S6. Flowchart of enrolled patients.
